# Low-Grade Esthesioneuroblastoma Presenting as SIADH: A Review of Atypical Manifestations

**DOI:** 10.1155/2012/582180

**Published:** 2012-12-04

**Authors:** Andrew Senchak, Judy Freeman, Douglas Ruhl, Jordan Senchak, Christopher Klem

**Affiliations:** ^1^Department of Otolaryngology, Walter Reed National Military Medical Center, 8901 Wisconsin Avenue Bethesda, MD 20889-5600, USA; ^2^Department of Pathology, Tripler Army Medical Center, Honolulu, HI 96859 5000, USA; ^3^Department of Otolaryngology, Tripler Army Medical Center, Tripler AMC, Honolulu, HI 96859 5000, USA; ^4^Grove City College, Grove City, PA 16127, USA

## Abstract

Esthesioneuroblastoma (ENB) is a neuroendocrine tumor that typically manifests as advanced stage malignancy in the superior nasal cavity. The hallmark symptoms include nasal obstruction and epistaxis, which result from local tissue invasion. Atypical clinical features can also arise and must be considered when diagnosing and treating ENB. These can include origin in an ectopic location, unusual presenting symptoms, and associated paraneoplastic syndromes. The case described here reports a nasal cavity ENB with atypical clinical features that occurred in a young female. Her tumor was low grade, appeared to arise primarily from the middle nasal cavity, and presented as syndrome of inappropriate antidiuretic hormone (SIADH). She also became pregnant shortly after diagnosis, which had implications on her surgical management. We review the atypical features that uncommonly occur with ENB and the clinical considerations that arise from these unusual characteristics.

## 1. Introduction 

Esthesioneuroblastoma is a rare nasal malignancy that accounts for 3% of all intranasal tumors [1]. A review of the world literature revealed up to 1000 published cases and suggested that the true incidence may be underestimated [2]. ENBs have a bimodal age of onset in the second and sixth decades of life and most commonly manifest as aggressive tumors in the superior aspect of the nasal cavity in close proximity to the cribriform plate [3]. The typical symptoms include unilateral nasal obstruction and epistaxis which present in later stages of disease, as early lesions are usually slow growing and asymptomatic [4]. 

ENBs arise from cells of neuroectodermal origin. In the nasal cavity, this type of tissue occurs in olfactory epithelium which can be found in the superior nasal cavity at the area of the cribriform plate, along the superior septum, and at the superior turbinate [5]. The pathologic features of this tumor are distinct and include nesting, low-grade stippled nuclei and neurofibrillary stroma with formation of pseudorosettes [6]. Like many malignancies, ENBs primarily cause invasion and destruction of surrounding structures, with potential for metastasis. 

We describe a patient with ENB that presented in several atypical ways—low-grade tumor which appeared to originate in the middle nasal cavity, presentation as syndrome of inappropriate antidiuretic hormone (SIADH), and diagnosis during pregnancy. We review the unusual characteristics that may uncommonly occur in ENBs and which must be considered when evaluating patients with this malignancy.

## 2. Case

A 28-year-old female presented with recurrent episodes of emesis, malaise, and diarrhea over a 3-year time period, from 2004 to 2007. She was admitted twice for hyponatremia, with a nadir serum sodium of 114 mEq/L. Her sodium levels normalized each time with saline administration, and so her hyponatremia was believed to be secondary to gastroenteritis and dehydration. In July 2007, she was seen again for malaise and recurrent emesis and was admitted with a sodium level at 112 mEq/L. Endocrinology was consulted for further workup. Their evaluation was significant for low calcium level (7.8 mg/dL), low serum osmolality (247 mOsm/kg), low urine osmolality (419 mOsm/kg), low phosphorus level (2.9 mg/dL), and low parathyroid hormone level (2.9 pg/mL). A repeat urine osmolality was elevated (1026 mOsm/kg). The remainder of her chemistry panel, urine sodium, cortisol, and thyroid function studies were normal. The endocrinology service diagnosed her with chronic SIADH and placed her on an oral course of demeclocycline to maintain sodium levels. 

### 2.1. Radiographic Studies

**Figure 1:**
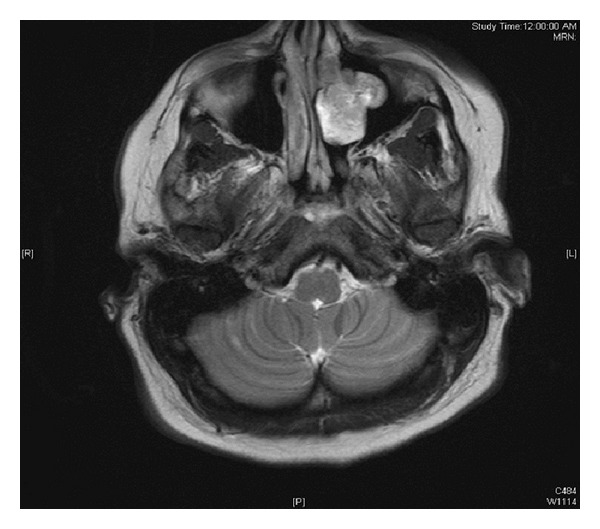
T2 weighted MRI with gadolinium showing enhancing mass in left lateral nasal wall and maxillary sinus.

**Figure 2:**
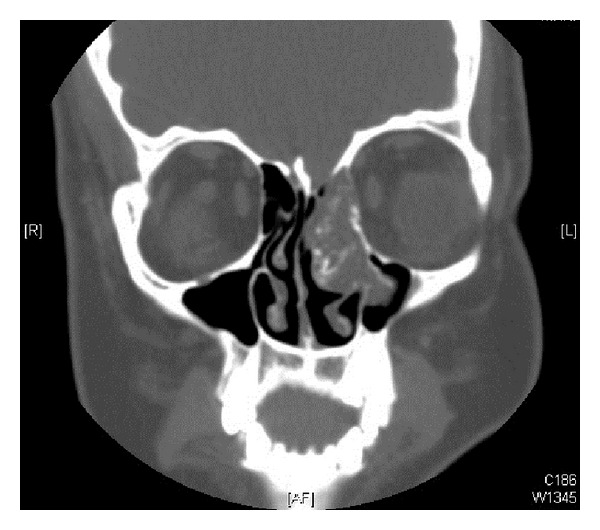
CT sinus demonstrating indolent mass with stippled calcifications centered at left middle turbinate.

An abdominal CT scan revealed bilateral nephrolithiasis without any evidence of neoplastic processes. A brain MRI with gadolinium demonstrated a heterogeneous mass in the left nasal cavity, with high T2 and low T1 signal, with no intracranial involvement (Figure 1). CT scan evaluation of the sinuses showed an indolent-appearing mass with stippled calcifications that was centered at the left ostiomeatal unit and middle turbinate, protruding into the left maxillary sinus, and extending superiorly toward the cribriform plate (Figure 2).

### 2.2. Surgical Treatment

The patient was referred to otolaryngology for evaluation of the nasal mass. Endoscopic evaluation revealed a 2 cm smooth-bordered, polypoid mass at the left ostiomeatal unit which extended superiorly and toward the nasal septum. Outpatient tissue biopsy of this mass was consistent with glial tissue, most likely glioma. This tumor was believed to be the underlying cause of the patient's SIADH, and surgical excision was recommended. During this time, however, the patient also discovered that she was 10-week pregnant. In consultation with the Obstetrics department, the decision was made to perform surgical excision because of the potential risk of recurrent hyponatremia throughout the pregnancy. During second trimester, the patient underwent endoscopic total resection of the neoplasm. The tumor was noted to emanate from the lateral nasal wall and dissected easily from the underlying bone. Although it extended superiorly to the cribriform area, there was no apparent attachment to the superior nasal cavity.

### 2.3. Pathological Evaluation

**Figure 3:**
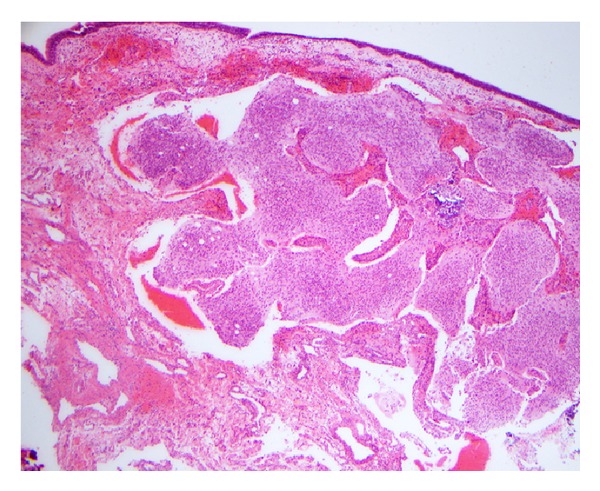
Low power view showing a nested proliferation of small hyperchromatic cells, prominent fibrillary background, focal microcalcification, and overlying respiratory epithelium.

Histologic examination of hematoxylin and eosin-stained slides demonstrated a proliferation of small, hyperchromatic cells exhibiting a predominantly nested pattern, in a background of fibrillar material with prominent dystrophic calcifications and a few pseudorosettes (Figure 3). The tumor was immunoreactive for chromogranin, glial fibrillary acid protein, pancytokeratin, neurofilament protein, neuron-specific enolase, Neu-N, S-100, and synaptophysin. The final pathology interpretation of the excised mass was consistent with grade 1 esthesioneuroblastoma with involvement of the middle turbinate remnant that was submitted as a margin. 

### 2.4. Followup

Following receipt of the pathology report, a wider surgical resection was recommended to the patient to ensure negative surgical margins given in the malignant diagnosis. The patient elected to wait until after delivery before proceeding with additional surgical management. In the interim, her sodium levels and osmolality normalized. Of note, her hypoparathyroidism and bilateral nephrolithiasis, which were felt to be due to repeated gastroenteritis, resolved as well. Approximately 8 months after her initial surgery, and following the uncomplicated delivery of her pregnancy, the patient underwent wider excision of the left lateral nasal cavity wall. All specimens from this second procedure were negative for residual malignancy. Her case was presented at a multidisciplinary tumor board, and the consensus opinion was that she would not require any adjuvant radiation or chemotherapy. The patient has been followed for 2 years since surgical treatment and has not demonstrated any recurrence of disease. Her sodium levels continue to be normal. 

## 3. Discussion

This case highlights several atypical manifestations that can occur with esthesioneuroblastomas. First, it is rare for an ENB to originate outside the region of the superior nasal cavity, where olfactory tissue is normally located. This disease is therefore a diagnosis of exclusion in ectopic locations. Several published reports confirm ectopic cases of ENB throughout the nasal cavity and the central nervous system. These include cases with primary origin in the posterior nasal septum [5], the nasal inferior meatus [7], the maxillary sinus [8, 9], the pterygopalatine fossa [10], the sella turcica [11], the pituitary gland [12] within the sphenoid sinus and petrous apex [13, 14], and in the dentoalveolar ridge [15]. Our patient had a low-grade tumor that appeared to emanate from the area of the left lateral nasal wall, although it did extend to the superior aspect of the nasal cavity. It is unknown whether ectopically located ENBs are associated with a more favorable tumor grade, and the reported cases range from low to high grade malignancies.

ENBs have also been reported to present with unusual signs and symptoms. For instance, some cases have occurred as bilateral noncontiguous tumors [16]. Some tumors invade into the orbit and can initially present with proptosis, sudden blindness, palsy of orbital cranial nerves, and ophthalmoplegia [17–20]. Compression and/or invasion of the nasolacrimal system has caused initial presentation with epiphora [21]. One case reports initial presentation as an oral cavity lesion, which resulted from direct extension from maxillary sinus involvement [8]. Psychological symptoms such as frontal lobe dysfunction and postpartum depression have been reported as presenting symptoms as well [22, 23]. Neck metastasis is known to occur in 10–30% of cases and can be the initial symptom of disease [24]. One case reports first presentation as bilateral metastatic cervical lymphadenopathy, which is very rare [17]. Since ENBs often demonstrate aggressive behavior and hematologic spread, recurrent disease can sometimes be first recognized in areas far away from the primary site. ENB metastasis most commonly occurs in lungs and bones, followed by liver, spleen, scalp, breast, adrenal gland, and ovary. Metastatic spread has also been reported in rare sites such as thoracic spine, spinal cord, parotid, and the trachea [17, 25, 26]. The heterogeneity of metastatic locations must be remembered when following a patient's clinical course even after ENB treatment. 

Very rarely, ENBs present with symptoms that are associated with paraneoplastic syndromes, such as our patient. These syndromes are known to affect up to 8% of all cancer cases, most often in small cell carcinoma of the lung, breast cancer, and gynecologic and hematologic malignancies [27]. Head and neck cancers are only infrequently involved. Paraneoplastic disorders arise when tumors produce hormones, peptides, or cytokines that lead to metabolic derangements. Antidiuretic hormone (ADH), adrenocorticotropic hormone (ACTH), calcium, and insulin represent the most common endocrine substances produced in paraneoplastic syndromes. We found 14 reports of ENB associated with production of ADH [8, 28–40] and 8 publications reporting ACTH production [41–48]. Sharma et al. report one fatal case of advanced ENB with humoral hypercalcemia of malignancy that became refractory to therapy [49]. One report describes an ENB that produced catecholamines, which normalized after surgery [40].

Although still infrequent, ACTH and ADH are the most common paraneoplastic processes to be associated with ENB. Ectopic ACTH production leads to elevated cortisol levels which usually results in Cushing syndrome. This syndrome consists of characteristic moon facies appearance, buffalo hump, and truncal obesity and can be associated with dysfunction in the cardiovascular, endocrine, neural, gastrointestinal, integumentary, and musculoskeletal systems. It is estimated that between 8% and 18% of total Cushing syndrome cases are due to ectopic ACTH production [50]. Most of the 8 reported cases of ectopic ACTH were from primary ENB tumors. It has also been found to first appear with recurrent disease, as in one pediatric case of late cervical lymph node recurrence [41] and in two adult cases of local recurrence despite aggressive surgical management [42, 48]. Although one patient died of recurrent disease, treatment of the ENB and/or recurrence led to resolution of the associated Cushing's syndrome in all other cases.

Excess of ADH production leads to a different clinical presentation due to the resultant hyponatremia and hyposmolality. These patients have an associated increase in urine osmolality due to elevated urinary sodium excretion and are usually euvolemic. Inappropriately high levels of ADH are also seen and are typically out of proportion to the hypoosmolality [37]. Other causes such as adrenal failure and hypothyroidism must be ruled out to establish a diagnosis of SIADH. Octreotide scanning can be used to look for areas of increased uptake, that indicate locations with neuroendocrine activity. Staining for arginine vasopressin (ADH) in the tumor tissue can also be done to help support the diagnosis. 

Development of SIADH does not appear to correlate with a higher cancer grade or stage in ENB cases. Two of the reported cases of SIADH occurred in low grade tumors, and one was mainly located around the middle turbinate, similar to our patient [37]. There have also been documented cases of SIADH that first occur in a locally recurrent tumor, without apparent paraneoplastic disease in the initial tumor [31]. Advanced stages can also be involved as one reported case occurred in a tumor that invaded into the cranial cavity [30]. One case occurred in an advanced stage ENB that transformed into a ganglioneuroma after chemoradiation therapy [32], and another one was in a mixed olfactory neuroblastoma/craniopharyngioma [34]. In all cases where tumor resection was possible, SIADH resolved after surgery, as in our patient. This strongly supports ENB as the cause of excess ADH production in these patients [27]. 

With most paraneoplastic processes, a diagnosis of cancer is usually made before discovery of ectopic hormone production [27]. In our patient, SIADH occurred before discovery of her tumor, and her hyponatremia was the impetus for obtaining the brain MRI. She also became pregnant during treatment, which made surgical resection more urgent. Development of hyponatremia can complicate the course of pregnancy, making treatment imperative. Initially, nausea and malaise develop from increased intracranial pressure as water migrates into cells. With increasing severity, seizures can develop with eventual coma and brainstem herniation in advanced stages [51]. Treatment with fluid restriction should be initiated, and early delivery should also be considered in cases of hyponatremia in pregnancy [52]. However, when ENB is suspected as the cause of SIADH, early discussions about the risks and benefits of surgery should be communicated between the patient, surgeon, anesthesia provider, and obstetrician. In cases where surgery is not performed, somatostatin analogues have been proposed to have a potential role in treatment, although this has not been adequately researched [37].

## 4. Conclusion 

While most ENBs originate in the superior nasal vault and present with typical symptoms of nasal obstruction and epistaxis in late stages of disease, uncommon manifestations should be considered as well. ENB should be kept in the differential of masses throughout the nasal cavity, paranasal sinuses, and surrounding anatomical locations. A patient with a history of prior ENB can have recurrence in distant locations as well. Paraneoplastic processes, particularly excess ACTH and ADH, should warrant radiographic workup for ENB. Unusual manifestations should be remembered in all cases of  ENB, as the stage and grade of tumor do not seem to demonstrate association with atypical disease.
